# Motile cilia and microvillar: accomplices of SARS-CoV-2 in penetrating mucus barrier and infecting airway epithelium

**DOI:** 10.1038/s41392-023-01387-7

**Published:** 2023-03-14

**Authors:** Peng Su, Fangfang Zhou, Long Zhang

**Affiliations:** 1 Center for Infection & Immunity of International Institutes of Medicine, the fourth affiliated hospital, ZheJiang University School of Medicine, YiWu, 322000 China; 2 Institutes of Biology and Medical Science, Soochow University, Suzhou, 215123 China; 3 Cancer Center, Zhejiang University, Hangzhou, China

**Keywords:** Infection, Infectious diseases

Recently in *Cell*, Chien et al.^1^ published their new findings on severe acute respiratory syndrome coronavirus 2 (SARS‑CoV‑2) penetrating through the mucosal barrier of respiratory by hijacking the cilia and microvilli of nasal epithelial cells, and elucidated the specific mechanism in detail using electron microscopy as well as phosphoproteomics. This study fully explained the way of SARS-CoV-2 breaking through the mucus barrier to infect nasal epithelial cells, and provided a novel direction for the blocking treatment of respiratory tract infections.

The culprit of coronavirus disease 2019 (COVID-19) pandemic, SARS-CoV-2, binds to angiotensin-converting enzyme 2 (ACE2) on host cell membrane with its spike protein (SP). The SP is then cleaved by host transmembrane serine protease 2 (TMPRSS2). Conformational changes of spike protein assist in bringing viral membrane closer to host cell membrane and subsequently facilitating their fusion, then viral genome is released into the host cell for replication.^2,3^ Upper airway is the major site of replication for SARS-CoV-2 and variant of concerns (VOCs) such as Omicron. Taking the nasal airway as an example, it is primarily composed of ciliated, basal, and mucus-secreting goblet cells. Epithelial cells are first covered by the periciliary layer (PCL), which contains mucin to prevent viral invasion. There is an additional mucus layer above the PCL, which traps pathogens and other particles preventing them from reaching the lungs.^4,5^ The mechanism by which SARS-CoV-2 breaks through the natural PCL barrier remains unclear.

Chien et al. aimed to determine the SARS-CoV-2 entry mechanism. To achieve this aim, they cultured primary human nasal epithelial cells (HNEs) in air-liquid interface (ALI) to induce the differentiation into nasal epithelial organoids. The authors inoculated SARS-CoV-2 D614G to nasal epithelial organoids at gradient MOI (MOI = 0.03, 0.3, and 3. MOI: multiplicity of infection). Immunofluorescence results showed that ciliated epithelial cells were the main target cells of infection. ACE2 and TMPRSS2 on motile cilia mediated viral invasion. More importantly, a small number (~3%) of ciliated epithelial cells were infected at early stage (~24 h post-infection (hpi)), while infected HNEs surged to ~80% at 48 hpi. This phenomenon suggested that SARS-CoV-2 infection of airway epithelial cells may occur in a two-step process.

Further experiments verified the two-step approach of infection. Treatment of HNEs with StcE, a kind of selective protease capable of disrupting mucin network, promoted initial viral invasion. This result demonstrated that mucin layer efficiently protects ALI-cultured HNEs from SARS-CoV-2 infection, which explains the delay of viral spread. Immunofluorescence and transmission electron microscopy (TEM) also revealed that virus particles were attached to cilia at 6 hpi, and 6% of cilia-attached viral particles were submerged in the PCL. Cilia depletion, using CEP83 shRNA, significantly inhibited SARS-CoV-2, respiratory syncytial virus (RSV), and human parainfluenza virus (PIV) infections. Blocking the retrograde trafficking of ciliary dynein, with the inhibitor Ciliobrevin D also effectively reduced positive ratio of ciliated cells at 48 hpi. These results suggested that cilia assisted SARS-CoV-2 in breaking through the mucosal barrier and invading the nasal epithelial cells.

Chien et al. further investigated the SARS-CoV-2 infection surge by monitoring virus replication and efflux at 24 and 48 hpi using immunofluorescence, TEM, and scanning electron microscope (SEM). Fluorescently labelled N protein was observed in cytoplasm at 6 hpi. From 24 hpi onwards, fluorescently labelled viral S and N protein colocalized with phalloidin labelled microvilli. A large number of newly generated virus particles accumulated in microvilli. Some large vesicles in cytoplasm, containing multiple progeny virus, were also driven near the microvilli. Further observations revealed that SARS-CoV-2 infection induced formation of dome-like alienated structures of microvilli, as well as a significant increase in highly extended microvilli. The phosphorylation level of the actin-binding protein ezrin (EZR), which is involved in microvilli formation, and the protein level of the microvilli core protein, EBP50, were significantly increased after infection. Before and after SARS-CoV-2 infection, using inhibitor NSC-668394 and SB-633825 targeting EZR and LOK/STK10 to treat HNEs, respectively, it was found that the inhibitor pretreatment had no effect. However, inhibitors administration at 18 hpi significantly reduced the number of infected cells at 48 hpi. It was concluded that following SARS-CoV-2 infection, the associated mediators involved in microvilli assembly process are over-activated, resulting in microvilli elongation and branching. Viral progeny shed by means of the alienated microvilli.

Chien et al. then cultured primary HNEs derived from patients with primary ciliary dyskinesia (PCD) and observed that at the later stage SARS-CoV-2 only infected localized areas rather than full coverage, forming positive plaques. The mucus flow triggered by external force increased the infected area and number of positive cells. This showed that mucus flow, based on ciliary beating, is important for the spread of viral progeny. To investigate the molecular mechanism by which SARS-CoV-2 infection affects microvilli biogenesis, Chien et al. used global phosphoproteomics and kinase set enrichment analysis (KSEA) to identify 72 over-activated kinases. Among them, the kinases involved in the regulation of microvilli and cytoskeleton were significantly activated, such as PAK1/4, AKT serine/threonine kinase (AKT1/2), mitogen-activated protein kinase P38 Alpha (p38), mitogen-activated protein kinase 3 (ERK1), and Rho-associated coiled-coil containing protein kinase 1 (ROCK1). Treatment of HNEs with PAK1/4 kinase inhibitor significantly reduced EZR phosphorylation in microvilli and effectively reduced infected ratio at 48 hpi. It has been suggested that PAK1/4 could be therapeutic targets for blocking SARS-CoV-2 spreading. The authors also compared the spatial and temporal infection patterns of SARS-CoV-2 and other VOCs in nasal epithelial organoids. In the Omicron group, 40% infected cells were observed at 24 hpi, which significantly advanced the transmission period of the viral progeny.

During natural evolution, humans have formed three immune barriers against pathogens, such as viruses and bacteria. The emergence of SARS-CoV-2 poses a severe challenge to the host’s antiviral immune function. Although there have been many reports on the mechanism of SARS-CoV-2 replication within host cells, the mechanism of SARS-CoV-2 penetrating respiratory mucus barrier remains unclear.

**Fig. 1:**
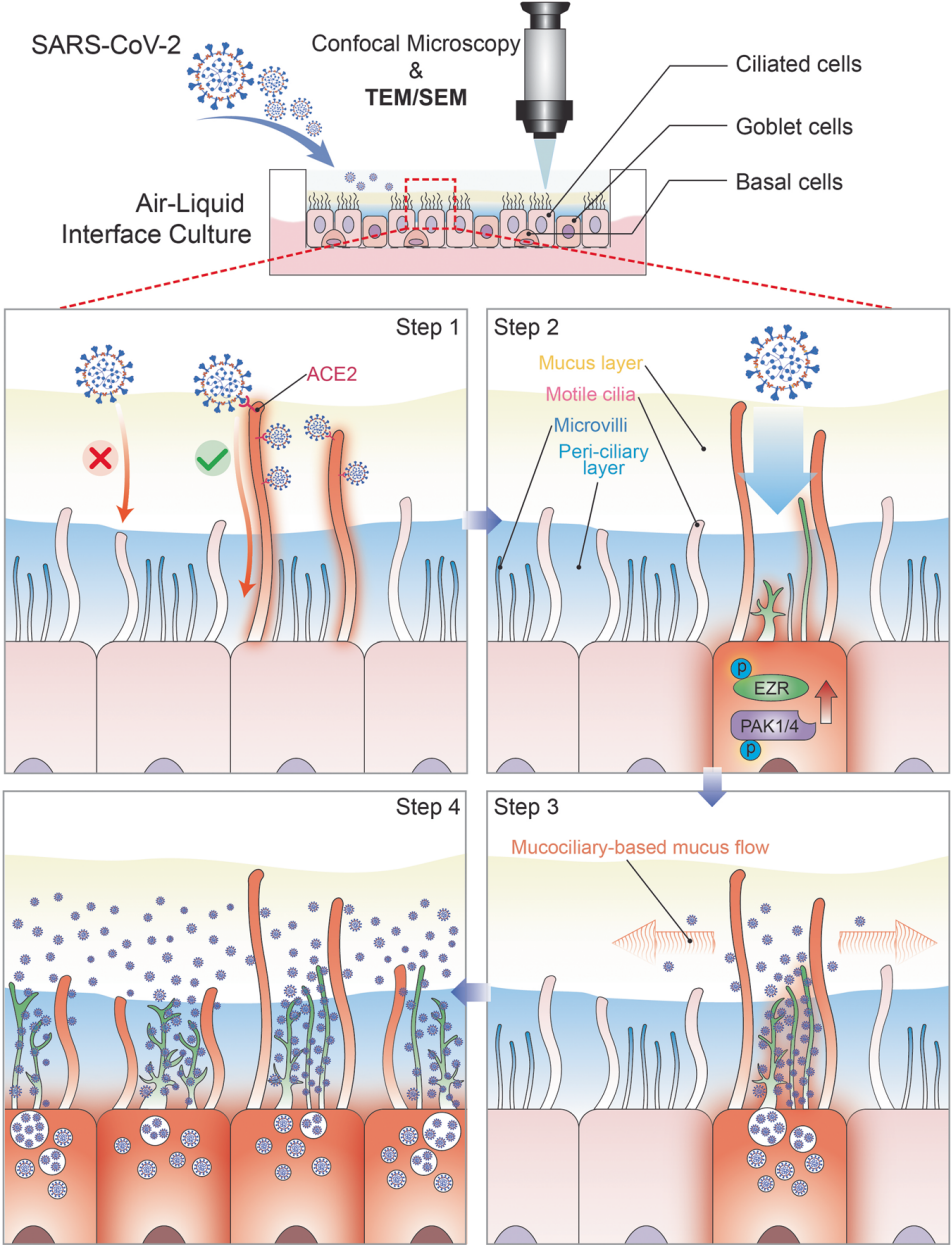
The step-by-step model of SARS-CoV-2 penetrating the mucus barrier and infecting human nasal epithelium. Air-liquid interface culture of primary human nasal epithelial cells to form nasal epithelial organoids composed of ciliated, goblet, and basal cells. Firstly, small amount of virus penetrates the peri-ciliary layer, along the cilia, after binding to ACE2 on cilia. Activation of various kinases such as PAK1/4 and phosphorylation of several actins such as EZR occur in the infected ciliated cells immediately. The microvilli then form dome-shaped alienated structures and high degree of extension. Cytoplasmic vesicle-encapsulated viral progeny pass through the PCL layer along alienated microvilli and exit at the mucus layer. Mucus flow, which depends on ciliary movement, assists the spread of viral progeny to other surrounding cells

This question was adequately addressed by Chien et al. using ALI-cultured nasal epithelial organoids for SARS-CoV-2 challenge. Their results showed that initially, only a small number of SARS-CoV-2 could penetrate the PCL through the airway cilia for efficient infection in trace amounts. Subsequently, SARS-CoV-2 hijacks the microvilli of infected cells to rearrange by promoting the activation of multiple kinases such as PAK1/4. Progeny virus exits via microvilli and spreads laterally to other areas via mucociliary transport (Fig. 1). This study fills the gap in the knowledge on initial stage of SARS-CoV-2 airway infection with solid data and reasonable conclusions. And it provides a potential therapeutic target that can be used to block the spread of the virus. It is of great significance to understanding the SARS-CoV-2 infection process and prevention and treatment of related diseases.

Chien et al. have revealed the specific steps and molecular mechanism of SARS-CoV-2 breaking the mucosal barrier at the early stage of airway infection through solid data. It is not difficult to find that the core of SARS-CoV-2 escape from mucociliary clearance is still the interaction between viral S protein and ACE2 receptor on the membrane of airway epithelial ciliary. Therefore, blocking or knocking down ACE2 receptors that perform non-essential physiological functions is also well worth trying as a blocking therapeutic strategy. Next researchers should further explore the physiological function of ACE2 on motile cilia and try to develop low-toxicity inhaled biologics to attenuate the escape effect of SARS-CoV-2 on mucociliary clearance by reducing the localization of ACE2 on motile cilia of airway epithelial cells. Alternatively, the design of liposome- or exosome-based low-irritation engineered vesicles capable of blocking cilia-ACE2 and residing in the airway mucosa for long periods of time is a worthwhile strategy to try. Combined with topical administration of kinase inhibitors to the nasal mucosa to modulate the function of microvilli, as mentioned by the authors, it may be possible to achieve treatment of different stages of SARS-CoV-2 infection.
